# Ciprofloxacin Removal via Acid-Modified Red Mud: Optimizing the Process, Analyzing the Adsorption Features, and Exploring the Underlying Mechanism

**DOI:** 10.3390/molecules29122928

**Published:** 2024-06-20

**Authors:** Jingzhuan Shi, Wanqiong Wang, Ziyi Li, Yingjuan Shi

**Affiliations:** 1 School of Chemistry and Environmental Science, Shaanxi University of Technology, Hanzhong 723001, China; wwq135792024@163.com (W.W.); 13109102007@163.com (Z.L.); 2 Shaanxi Reconnaissance Design & Research Institute of Water Environmental Engineering, Xi’an 710021, China; sxssltshzhfyc@163.com

**Keywords:** acidified red mud, ciprofloxacin, adsorption, response surface methodology

## Abstract

In this study, RM (red mud) was acidified with sulfuric acid, and the acidified ARM (acidified red mud) was utilized as an innovative adsorption material for treating antibiotic-containing wastewater. The adsorption conditions, kinetics, isotherms, thermodynamics, and mechanism of ARM for CIP (ciprofloxacin) were investigated. The characterization of the ARM involved techniques such as scanning electron microscopy (SEM), transmission electron microscopy (TEM), Brunauer–Emmett–Teller (BET), X-ray diffraction (XRD), X-ray fluorescence (XRF), thermogravimetric analysis (TGA), and NH_3_-TPD analysis. Adsorption studies employed a response surface methodology (RSM) for the experimental design. The results showed that ARM can absorb CIP effectively. The RSM optimal experiment indicated that the most significant model terms influencing adsorption capacity were solution pH, CIP initial concentration, and ARM dosage, under which the predicted maximum adsorption capacity achieved 7.30 mg/g. The adsorption kinetics adhered to a pseudo-second-order model, while equilibrium data fitted the Langmuir–Freundlich isotherm, yielding maximum capacity values of 7.35 mg/g. The adsorption process occurred spontaneously and absorbed heat, evidenced by Δ*G*^θ^ values between −83.05 and −91.50 kJ/mol, Δ*S*^θ^ at 281.6 J/mol/K, and Δ*H*^θ^ at 0.86 kJ/mol. Analysis using attenuated total reflection Fourier-transform infrared spectroscopy (ATR-FTIR) indicated a complex reaction between the Al–O in the ARM and the ester group –COO in CIP. The C=O bond in CIP was likely to undergo a slight electrostatic interaction or be bound to the internal spherical surface of the ARM. The findings indicate that ARM is a promising and efficient adsorbent for CIP removal from wastewater.

## 1. Introduction

Red mud (RM) is a by-product of bauxite ore processing with caustic soda during alumina manufacturing [1,2]. It was estimated that RM reached 3.5 × 10^9^ tons worldwide in 2014 [3]. In China, the annual yield of RM is 7 × 10^7^ tons and the accumulated inventory is about 6 × 10^8^ tons [4]. This large accumulation of RM has caused serious environmental problems. Currently, the primary method for RM disposal is dam construction, while secondary contamination induced by RM’s high alkalinity occurs frequently [5,6]. Therefore, public concern about RM utilization has grown [7]. So far, various attempts have been made to exploit RM, including the production of building materials, the recycling of rare metals, and the preparation of RM-based catalysts and adsorbents [8,9,10,11]. Nevertheless, the adsorption performance of RM has mainly been focused on the removal of heavy metal ions, organic dyes, and phosphorus [12,13,14]. To the best of our understanding, there has been limited research on RM’s capacity to adsorb antibiotic contaminants.

Fluoroquinolone antibacterial agents (FQs), a class of powerful synthetic antibiotics, are extensively utilized in both human and veterinary healthcare [15]. Their pervasive use raises concerns as FQs can infiltrate aquatic ecosystems through various pathways, including effluents from pharmaceutical manufacturing, sewage sludge, and agricultural waste. FQs are increasingly found in various water bodies. Notably, studies in the U.S. identified four FQs—ciprofloxacin, norfloxacin, enrofloxacin, and sarafloxacin—with average concentrations reaching up to 0.12 μg/L in surface waters [16]. Furthermore, ciprofloxacin has been found in hospital wastewater at concentrations between 19 and 155 µg/L, indicating a significant potential for environmental contamination [17]. Even at low levels, the presence of these antibiotics in water poses a risk to both ecosystems and human health, with some contributing to bacterial genotoxicity in sewage [18]. To mitigate this risk, methods such as ozonation, photocatalysis, Fenton oxidation, and adsorption have been explored for FQ removal from water, with adsorption particularly favored for its efficiency, ease of implementation, and cost-effectiveness [19].

In this study, we treated RM with sulfuric acid to create an adsorbent called ARM for extracting CIP from water. The morphology, structure, and properties of RM and ARM were analyzed using XRD, BET-BJH, SEM, TEM, XRF, TG-DSC, ATR-FTIR, and NH_3_-TPD; compared with RM, ARM has a significantly improved adsorption capacity for CIP. Because the acid changed the strong alkalinity, increased the specific surface area, and changed the internal structure of the RM, the Fe ion content increased. We employed RSM utilizing a Box–Behnken design to fine-tune the adsorption parameters, including temperature, pH, initial CIP concentration, and ARM amount. This study also explored the adsorption kinetics, equilibrium isotherms, and thermodynamics associated with CIP removal via ARM. The adsorption process occurred spontaneously and absorbed heat. Furthermore, the adsorption mechanism was analyzed using ATR-FTIR to provide a scientific basis for the comprehensive utilization of RM and the remediation of ciprofloxacin-polluted water.

## 2. Results and Discussion

### 2.1. Adsorption Property of RM and ARM

**Figure 1:**
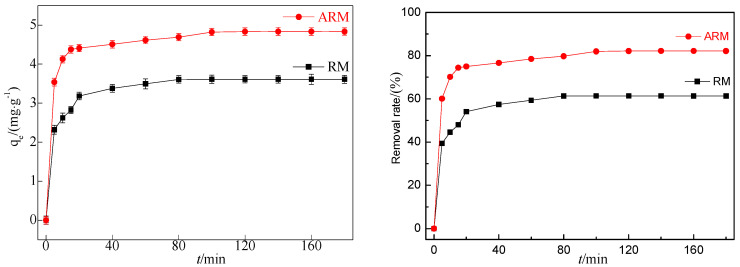
Effect of adsorption by RM and ARM (pH_0_ = 3.04, T = 45 °C, [CIP] = 20 mg/L, [RM] = 3.4 g/L, [ARM] = 3.4 g/L, r = 250 rpm).

According to Figure 1, under the same conditions, the adsorption capacity of CIP by RM after sulfuric acid acidification increased from 3.62 to 4.84 mg/g, and the removal rate increased from 61.41% to 82.21%. This indicates that ARM has a significantly better adsorption performance for CIP than RM.

### 2.2. Characteristics of RM and ARM

#### 2.2.1. Morphology of RM and ARM by SEM and TEM

**Figure 2:**
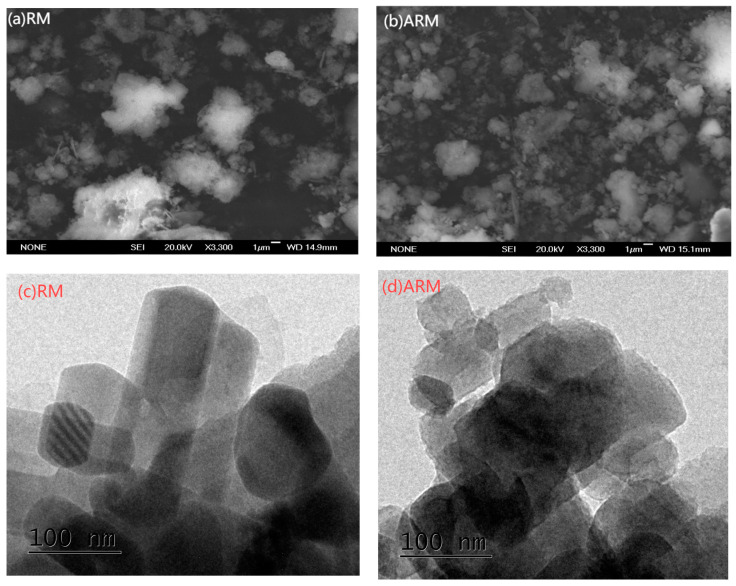
SEM images of RM (**a**) and ARM (**b**), and TEM images of RM (**c**) and ARM (**d**).

SEM was utilized to assess the surface and structural characteristics of the RM samples. Figure 2a shows that RM consists of spherical or flaky particles of micron size. Variations in particle size may result from the presence of both unchanged and newly formed minerals within the bauxite ore. The larger particles could be either the original minerals in the bauxite or aged mineral oxides [20]. It is evident in Figure 2b that ARM particles exhibit a broad spectrum of sizes and possess irregular shapes. These changes in size and morphology might be due to the dissolution of some minerals throughout the acidification process [21].

TEM analysis results indicated that the round aggregates of RM (Figure 2c) are composed of fine particles and scattered irregular quadrilateral crystals and are mainly porous frames with cemented connections, without a definite shape [22]. These may be crystallized mineral phases and newly formed mineral phases in bauxite that may be calcite, sodalite, silicates, and iron oxides [23].

Figure 2d shows that RM is acidified, the internal crystal structure changes, and the crystal surface is corroded by acid. Acidic surface treatments lead to the development of microspores or mesopores on the RM surface and create localized adsorption sites on the RM surface. This is the reason why acidic treatments could result in a greater specific surface area and porosity [24]. This result was consistent with the BET-BJH analysis.

#### 2.2.2. BET-BJH Analysis

Figure S1 displays the nitrogen adsorption and desorption curves for both RM and ARM. Both belong to type III (the curve has no inflection point and is convex downward throughout the pressure range). The surface areas of RM and ARM as measured by BET were calculated to be 10.96 and 17.44 m^2^/g, respectively. ARM presents a larger specific surface area and thus is more readily able to adsorb CIP.

**Figure 3:**
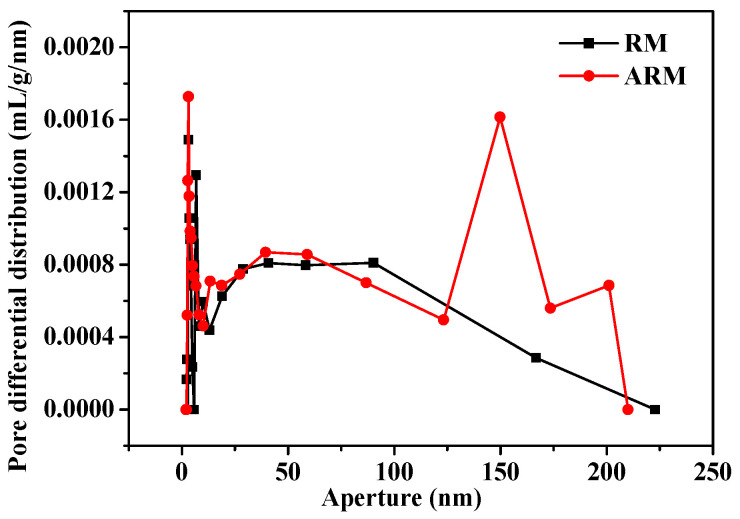
Pore size distribution of RM and ARM.

Figure 3 illustrates the pore size distributions for both RM and ARM. RM contains a small number of micropores and simple open porous channels. By contrast, the ARM contains not only micropores but also larger mesopores and macropores (micropores < 2.0 nm; smaller mesopores 2.0–10.0 nm; larger mesopores and macropores ≥ 10 nm) [25], and the channel structure is very complex, with a peak appearing at 150 nm. The average pore sizes of RM and ARM were 40.93 and 45.41 nm, respectively. This indicates that acidification treatment contributed to the dissolution of aluminum, sodium, and/or silica compounds within RM and the primary surface structure of RM changed; thus, the porous channel widths increased.

#### 2.2.3. XRD and XRF Analysis

**Figure 4:**
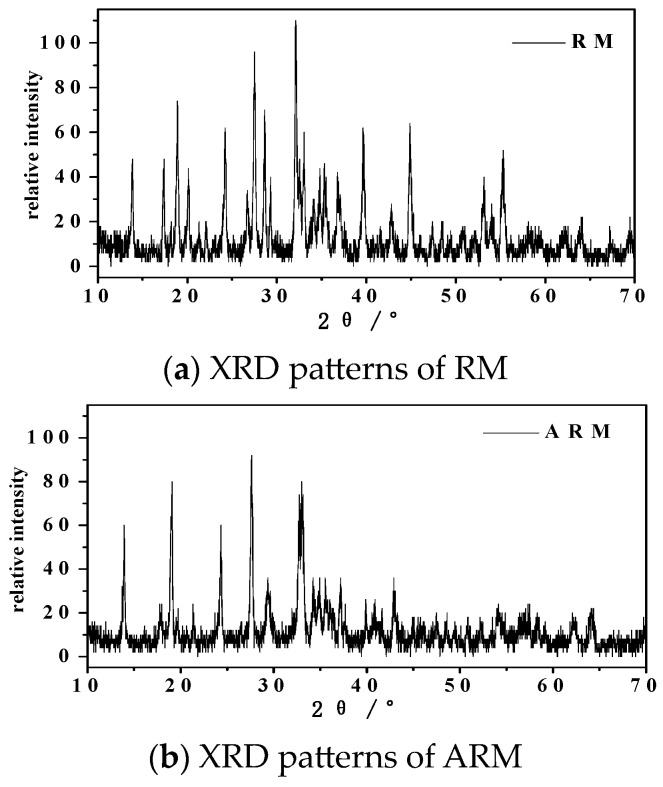
XRD patterns of RM (**a**) and ARM (**b**).

As depicted in Figure 4, XRD analysis revealed that the predominant components of the RM samples are sodalite (S) (Na_6_(Al_6_Si_6_O_24_)CO_3_), hematite (H) (Fe_2_O_3_), gibbsite (G) (Al(OH)_3_), boehmite (B) (γ-AlO(OH)), TiO_2_ (anatase and rutile), and quartz (SiO_2_). The broad peaks observed in the XRD pattern are consistent with the findings reported by Guru et al. [26].

The XRF analysis results are shown in Table 1, indicating that after the acidification treatment of RM, the CaO content rose from the original 26.01% to 40.13%. Iron oxide was a frequently encountered active constituent in catalysts [27], Fe_2_O_3_ content rose from 11.56% to 28.37%. This phenomenon may be due to the fact that the crystals in RM dissolved under the action of the acid, producing more iron oxide. The reduction in the calcite phase within ARM is attributed to dissolution, leading to the generation of a more reactive surface conducive to CIP adsorption onto ARM [28]. Concurrently, the presence of Al_2_O_3_, SiO_2_, and TiO_2_ after modification could be attributed to their role as carriers [29].

#### 2.2.4. TG-DSC Analysis

As depicted in Figure S2a, the total weight loss of RM from 24 °C to 1100 °C was 13.73%. The loss of 3% in the first stage from room temperature to 300 °C was mainly due to the mass loss of adsorbed water in the red mud and internal structure water volatilization and partial hydroxide decomposition. For example, Al(OH)_3_ was decomposed into AlO(OH), part of AlO(OH) was further decomposed into Al_2_O_3_, and part of FeOOH was decomposed into Fe_2_O_3_ [30]. In the second stage, the weight loss from 300 °C to 600 °C was 6.6%, mainly due to the mass loss of the further decomposition of hydroxide. In the third stage, the weight loss from 600 °C to 1100 °C was 4.1%, mainly due to the mass loss of CO_2_ released by the thermal decomposition of calcium nepheline in red mud.

As depicted in Figure S2b, the total weight loss of ARM from 24 °C to 1100 °C was 15.55%. In the first stage, the weight loss from room temperature to 300 °C was 3.8%, mainly due to the adsorption water of red mud and the evaporation of internal structural water. The weight losses of 7.3% and 4.4% in the second stage at 300~600 °C and the third stage at 600~1100 °C, respectively, were due to the loss of gas released by the thermal decomposition of hydrated garnet and calcium nepheline in the red mud.

The results of the TG analysis are compared with literature data [31] in Table S3. The results show that the changes in RM and ARM were caused by the internal structure of crystal water, which has a lower water content. When the ARM was heated, no obvious chemical or physical reaction occurred, and the thermal stability was strong. The TG trends of RM and ARM are consistent with Zhu’s research results [32].

#### 2.2.5. NH_3_-TPD Analysis

**Figure 5:**
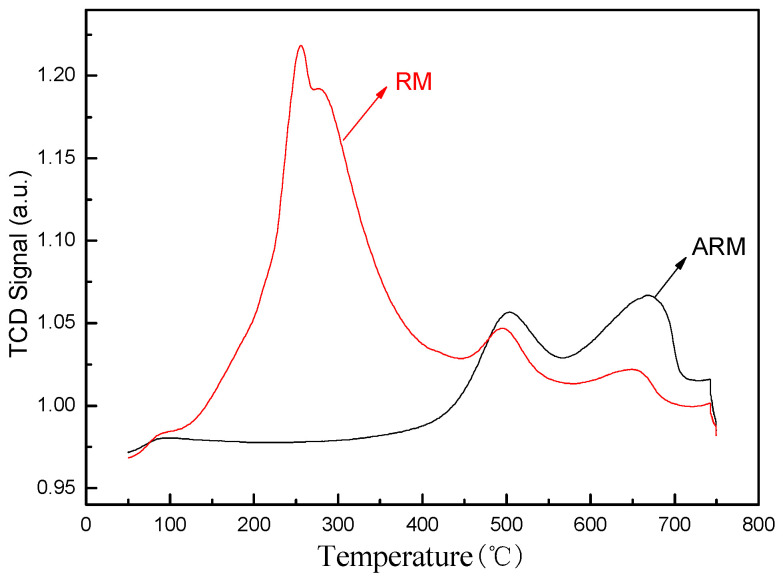
NH_3_-TPD of RM and ARM.

The experimental results of NH_3_-TPD are shown in Figure 5. As we all know, the adsorption capacity of the adsorbent is related to the number of active sites, the oxidation state of the active phase, the mobility of oxygen in the adsorbent matrix, and other factors [33]. Peaks in the low-temperature range of 100~300 °C indicate that the physical adsorption of molecular oxygen and chemisorbed oxygen are desorbed above or near the surface of the adsorbent, peaks in the 300~500 °C range are attributed to the surface lattice oxygen in the adsorbent, and peaks in the high-temperature range of 500~800 °C are attributed to the bulk lattice oxygen of the adsorbent. The peak intensity of RM at about 245 °C is extremely high, which is mainly the signal value generated by the decomposition of the CaCO_3_ in red mud into CO_2_ at this temperature. In addition, RM has two small peaks at 495 °C and 650 °C, corresponding to the desorption of surface oxygen vacancies and bulk oxygen vacancies. The peak of ARM appears at 500 °C and 668 °C, respectively. Compared with RM, which shifted toward a high temperature, the peak strength increased, indicating that the interaction between sulfuric acid and Fe changed the electronic structure of the oxygen vacancy of the adsorbent. This resulted in surface defects, providing a suitable adsorption site for oxygen molecules and strengthening the adsorption capacity of oxygen species on the adsorbent.

### 2.3. Optimization of Process Variables Using RSM

#### 2.3.1. Establishment of the Model

Response surface optimization for the adsorption conditions of CIP on RSM was performed according to the Box–Behnken method. The experimental design results are shown in Table S4.

Under the different adsorption conditions designed in the test, the measured adsorption capacity of CIP varied from 1.04 to 7.23 mg/g. The results in Table S5 were further subjected to multiple regressions and variance analysis employing Design Expert 10.7. The results, along with the corresponding data, are summarized in Table S5. Equation (1) presents a quadratic polynomial model representing adsorption capacity, incorporating four independent variables.

*Y* = 2.34 − 0.046*A* − 1.28*B* + 1.25*C* − 0.62*D* − 0.13*AD* − 0.71*BC* + 0.35*BD* − 0.17*CD* + 1.23*B*^2^ − 0.11*C*^2^ − 0.20*D*^2^ (1)

where

*Y*—the response variable (adsorption capacity) and the actual values of the predictors;

*A*—reaction temperature (°C);

*B*—solution pH;

*C*—CIP initial concentration (mg/L);

*D*—ARM dosage (g/L).

As shown in Table S5, the *p* values of the parameters (involving *B*, *C*, *D*, *BC*, and *B*^2^) were all lower than 0.05. This implied that the solution pH, CIP initial concentration, ARM dosage, interaction between pH and CIP initial concentration, and the effect of squares of pH all showed significant influences on the adsorption capacity. The *p* values of other parameters were larger than 0.05, indicating that the effect of other factors on adsorption capacity was insignificant. Moreover, the high *F* value (*F*_model_ = 40.08), and the associated very low probability value (*p* < 0.0001) of the model suggest statistical significance, indicating a well-fitted model.

**Figure 6:**
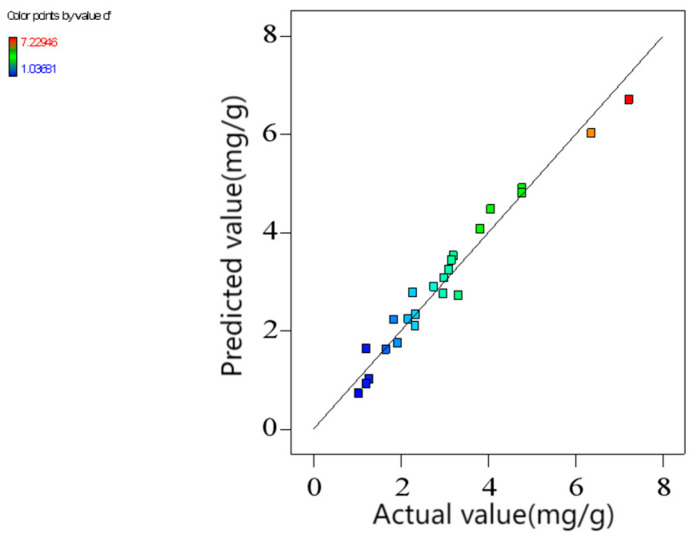
Comparison of the predicted and actual adsorption capacities (pH_0_ = 3.04, T = 45 °C, [CIP] = 30 mg/L, [ARM] = 3.4 g/L, r = 250 rpm).

Figure 6 shows the adsorption capacities calculated from the established quadratic polynomials and the measured ones. It was obvious that both the predicted and the measured values conformed to a normal distribution, with the coefficient of determination *R*^2^ = 0.9629, indicating that 96.3% of all variation can be explained. Furthermore, the adjusted coefficient of determination (*R*^2^_Adj_) for the model stood at 0.9389, indicating a strong fit. The signal-to-noise ratio (SNR) was 25.92, much higher than 4, so the model is highly reliable, and the data are reasonable. Therefore, Equation (1) accurately and reasonably reflects the relationship between the adsorption capacity and the variables.

#### 2.3.2. Response Surface Analysis

Graphical representations of the models aid in comprehending the impacts of experimental variables on the responses. As mentioned, the interaction between solution pH and CIP initial concentration was significant. Figure S3 displays both the 3D surface graph and the corresponding contour plot illustrating the relationship between pH and CIP initial concentration. It is evident that adsorption capacity rose with increasing CIP initial concentration but decreased as the solution pH decreased, which was attributed to different dissociation forms of CIP at different pH levels. The CIP was mainly dissociated as H_4_CIP^3+^ at a low pH value. The chemical activity of H_4_CIP^3+^ was stronger than H_3_CIP^2+^ and was more favorably adsorbed by the active materials in ARM [34]. When the solution was alkaline, the active components in ARM were more readily precipitated, thus reducing the adsorption ability of ARM for CIP.

#### 2.3.3. Optimization Analysis

Numerical optimization was employed to determine the optimal conditions for CIP adsorption. Based on the fitted model, the optimal adsorption parameters were identified: a temperature of 45 °C, a pH of 3.04, an initial CIP concentration of 29.20 mg/L, and an ARM dosage of 3.40 g/L, resulting in an adsorption capacity of 7.30 mg/g.

### 2.4. Adsorption Kinetics

We utilized the pseudo-first-order and second-order kinetics, represented by Equations (2) and (3), to elucidate the sorption mechanism of CIP on ARM. Additionally, we employed the intraparticle diffusion model, Equation (4), to elucidate the diffusion mechanism.

(2) $$\ln(q_{e}-q_{t})=\ln(q_{e})-k_{1}t$$

(3) $$\frac{t}{q_{t}}=\frac{1}{q_{e}}t+\frac{1}{k_{2}{q_{e}}^{2}}$$

(4) $$q_{t}=k_{i}t^{1/2}+C$$

where

*q*_e_—the adsorption capacities under adsorption equilibrium (mg/g);

*q*_t_—the adsorption capacities at time *t* (mg/g);

*k*_1_—the rate constant of the pseudo-first-order kinetic model (min^−1^);

*k*_2_—the rate constant of the pseudo-second-order kinetic model (g^−1^·min^−1^);

*k*_i_—intraparticle diffusion constant [mg/(g·min^1/2^)];

*C*—a constant expressing the extent of dominance of intraparticle diffusion (mg/g).

**Figure 7:**
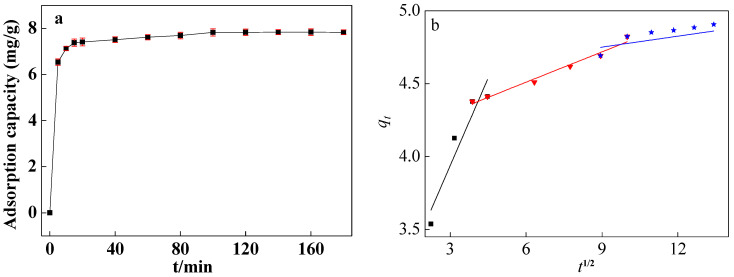
Adsorption capacity of ARM for CIP (**a**) and fitting curves of intraparticle diffusion models (**b**) (pH_0_ = 3.04, T = 45 °C, [CIP] = 30 mg/L, [ARM] = 3.4 g/L, r = 250 rpm).

The fitting outcomes are presented in Figure 7 and Figure S4 and Table 2. Notably, the model plot for pseudo-second-order kinetics closely aligned with the experimental data (Figure S4a), boasting a correlation coefficient (*R*^2^) of 0.9998. The results showed that ARM adsorption of CIP was controlled by chemical adsorption, and the reaction rate constant was dictated by the square of the number of unoccupied adsorption sites on the adsorbent surface [35]. The pseudo-first-order kinetic model displayed a deviation from the experimental points, yielding an *R*^2^ value of 0.88301 and a maximum adsorption capacity of 4.17 mg/g.

The intraparticle diffusion model (Figure 7b) demonstrated that the whole adsorption process involved rapid adsorption, slow adsorption, and intraparticle diffusion. The fitting curve does not pass through the origin, which further implied that other control processes existed [36].

### 2.5. Adsorption Isotherm

The Langmuir, Freundlich and Langmuir–Freundlich isotherm models were deployed to analyze the sorption behavior of CIP toward ARM. The Langmuir model (Equation (5)) posits monolayer adsorption on homogeneous surfaces, and the Freundlich isotherm (Equation (6)) characterizes multilayer adsorption on heterogeneous surfaces. The Langmuir–Freundlich model (Equation (7)), amalgamating features from both Langmuir and Freundlich isotherms, incorporates three parameters into an empirical equation, rendering it more adept at delineating the extent of mono or multilayer adsorption within a system.

(5) $$\frac{C_{e}}{q_{e}}=\frac{C_{e}}{q_{m}}+\frac{1}{K_{L}q_{m}}$$

(6) $$\ln q_{e}=\ln K_{F}+\frac{1}{n}\ln C_{e}$$

(7) $$q_{e}=\frac{K_{\mathrm{LF}}q_{m}{C_{e}}^{n}}{1+K_{\mathrm{LF}}{C_{e}}^{n}}$$

where

*C*_e_—the solute equilibrium concentration (mg/L);

*q*_e_—equilibrium adsorption capacity (mg/g);

*q*_m_—theoretical maximum monolayer adsorption capacity (mg/g);

*K*_L_—the Langmuir adsorption constant related to adsorption energy (L/mg);

*K*_F_—the Freundlich constant related to removal efficiency of solute (L/g);

*n*—constant characterizing the adsorption strength;

*K*_LF_—the Langmuir–Freundlich constant (L/mg).

**Figure 8:**
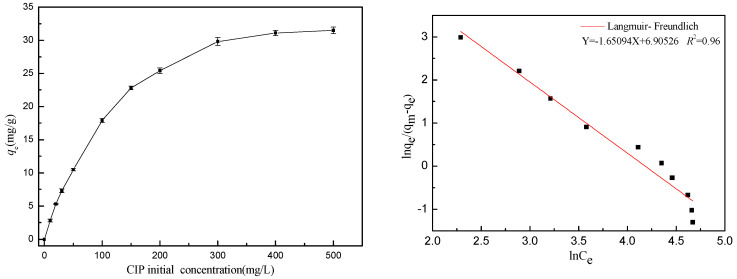
Fitting curves of the Langmuir–Freundlich isotherm model (pH_0_ = 3.04, T = 45 °C, [CIP] = 10~500 mg/L, [ARM] = 3.4 g/L, r = 250 rpm).

The isotherm fitting outcomes (Figure 8) alongside the error parameters and *R*^2^ values (Table 3) indicated that all three isotherm models provided acceptable fits for the experimental data. Notably, the Freundlich and Langmuir–Freundlich models exhibited a superior fit compared to the Langmuir model, suggesting that the adsorption process involved a combination of mono and multilayer adsorption. This implies that real adsorption took place on heterogeneous surfaces with varying adsorption sites, facilitated by multiple adsorption interactions. According to the Langmuir–Freundlich fitting results, the maximum adsorption capacity of CIP toward ARM increased with temperature, being 7.35 mg/g at 45 °C, which conformed to the measured maximum adsorption capacity (7.84 mg/g).

Additionally, as depicted in Table 4, we conducted a comparative analysis of our adsorbents with those reported in the literature. Adsorbents with a strong adsorption capacity generally come from the preparation of emerging materials. As a kind of solid waste, RM has the characteristics of being a cheap raw material and simple to modify, and the adsorption capacity of acidified red mud for CIP is higher than sodium alginate and kaolinite. These findings underscore the considerable potential of ARM as an antibiotic adsorbent.

### 2.6. Adsorption Thermodynamics

Based on the Van’t Hoff equation (Equations (8)–(10)), Δ*G*, Δ*H*, and Δ*S* can be calculated according to the adsorption constants of the Langmuir–Freundlich isotherm at different temperatures:

(8) $$\Delta G=-RT\ln K_{\mathrm{LF}}$$

(9) ΔG=ΔH−TΔS

(10) $$\ln K_{\mathrm{LF}}=-\frac{\Delta H}{RT}+\frac{\Delta S}{R}$$

where *K*_LF_ represents the Langmuir–Freundlich coefficient of adsorption equilibrium.

The experimental equilibrium constant should be converted to the standard equilibrium constant by the standard state [47]. The standard equilibrium constant *K*_LF_^θ^ of Equation (10) is:

(11) $${K_{\mathrm{LF}}}^{\theta }=K_{\mathrm{LF}}{\left(C^{\theta }\right)}^{n_{\mathrm{LF}}}$$

where *K*_LF_^θ^ represents the standard equilibrium constant of *K*_LF_; *C*^θ^ represents the standard states of solution in liquid, and *C*^θ^ = 1 mol/L.

According to Equations (12) and (13), *K*_LF_^θ^ can be used to calculate the Δ*G*^θ^, Δ*H*^θ^, and Δ*S*^θ^.

(12) $$\ln{K_{\mathrm{LF}}}^{\theta }=-\frac{\Delta H^{\theta }}{RT}+\frac{\Delta S^{\theta }}{R}$$

(13) $$\Delta G^{\theta }=\Delta H^{\theta }-T\Delta S^{\theta }$$

**Figure 9:**
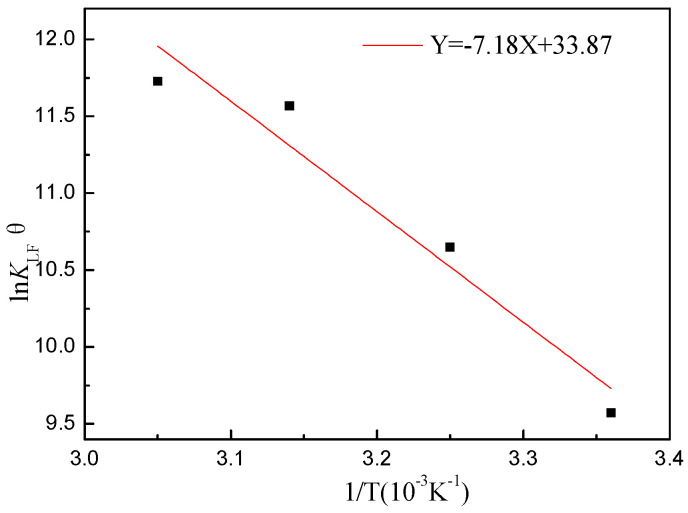
Van’t Hoff linear graph (pH_0_ = 3.04, T = 25~55 °C, [CIP] = 10~500 mg/L, [ARM] = 3.4 g/L, r = 250 rpm).

Figure 9 displays a plot of ln *K*_LF_^θ^ versus 1/*T*. From the slope and intercept of the straight line, Δ*G*^θ^, Δ*H*^θ^, and Δ*S*^θ^ were calculated, and the results are shown in Table 5. Δ*G*^θ^ was negative, indicating that the adsorption capacity of CIP toward ARM was a spontaneous process. Δ*G*^θ^ decreased from −83.05 to −91.50 kJ/mol with a temperature increase from 25 °C to 55 °C. This implies that the adsorption performance of the adsorbent strengthened with temperature [48]. In addition, the positive Δ*H*^θ^ indicates that the adsorption process involved an endothermic reaction [49].

### 2.7. ATR-FTIR Analysis of CIP and ARM

**Figure 10:**
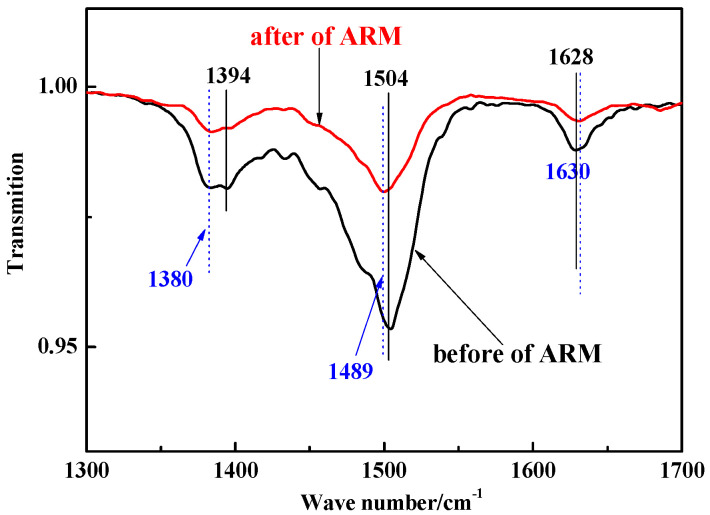
FTIR spectra of CIP and ARM before and after CIP adsorption (black indicates acidified red mud (before adsorption); red indicates acidified red mud (after adsorption)).

The FTIR spectra of ARM before and after absorbing CIP are shown in Figure 10.

In the case of ARM, the band observed at 1628 cm^−1^ originated from the stretching vibration of the Fe–O bond (hematite), whereas the bands at 1394 and 1504 cm^−1^ were attributed to the stretching vibration of the Al–O bond (boehmite) and the bending vibration of the O–H bond, respectively. These findings suggest the presence of adsorbed water within ARM [28].

After adsorbing CIP, the characteristic bands of the Al–O bond shifted from 1394 to 1380 cm^−1^, which was attributed to a complex reaction between the Al–O in ARM and the –COO in CIP [50]. The O–H bonds at 1504 cm^−1^ shifted, implying that the adsorbed water in ARM was consumed [51]. The Fe–O bond at 1628 cm^−1^ shifted (albeit to no significant extent), indicating that the C=O bond in CIP underwent a slight electrostatic interaction with the Fe–O bond in ARM or was bound to the internal spherical surface of the ARM [52].

### 2.8. Adsorption Stability of ARM

To investigate the stability of ARM for CIP adsorption, different types of water with different pH levels were selected as the leaching solutions to wash the adsorbed ARM. The experiment is shown in Figure S5.

As shown in Figure S5a, the leaching rate of CIP decreased with increasing pH. When the pH increased from 3 to 11, the corresponding leaching rate decreased from 20.59% to 10.92%. Compared with alkaline environments (pH = 7–11), ARM with saturated adsorption exhibited poorer adsorption stability in acidic environments (pH = 3–5). This is because at pH = 3–5, H+ competes with H_4_CIP^3+^, leading to an increase in the concentration of H_4_CIP^3+^ in the leachate and a decrease in the stability of ARM.

As shown in Figure S5b, with a pH ≈ 7, using ultra-pure water (UP), lake water (LW), and sewage water (SW) as leachate, the leaching rates of CIP were 12.30%, 10.29%, and 8.93%, respectively. The stability was as follows: SW > LW > UP. Therefore, ARM exhibited higher stability in sewage water and lake water.

## 3. Material and Methods

### 3.1. Materials

RM was obtained from Shanxi Aluminum Corporation in Yuncheng City, Shanxi Province, China; the pH value of the leaching solution was 11.04. CIP, with a purity exceeding 98%, was acquired from Tokyo Kasei Kogyo Co., Ltd. (TCI, Tokyo, Japan), with a molecular structure of C_17_H_18_FN_3_O_3_. The remainder of the chemicals used were of analytical quality, and all solutions were formulated using Millipore’s ultra-pure water (Billerica, MA, USA).

### 3.2. Methods

#### 3.2.1. Preparation of ARM

Before acid treatment, RM underwent drying and was then finely milled to a size allowing it to sieve through a 150-mesh (0.100 mm) screen. Then 50 g pre-treated RM was weighed and placed into a 1.0 L beaker, into which water was added under stirring until a liquid suspension was formed. The liquid–solid ratio of H_2_SO_4_ and RM was 0.5 mL/g, and the pH of the suspension was adjusted with H_2_SO_4_ to 3.4 after stirring at 100 r/min for 12 h. After standing, the supernatant of the mixture was rinsed with water repeatedly until its pH became neutral. Finally, the solid residues were collected through vacuum filtration, dried at 100 °C, and sieved through a 150-mesh screen for use.

#### 3.2.2. Measurement of Adsorption Capacity

A certain amount of ARM (0.6, 0.8, or 1.0 g) was added into a 250 mL brown conical flask that contained 200 mL of a known concentration of CIP. The initial CIP concentrations tested were 10, 20, and 30 mg/L. The pH of the mixture was set to targeted levels (3.0, 5.0, 7.0, 9.0, or 11.0 ± 0.2) using 0.1 mol/L NaOH or HCl. Afterward, the flask was sealed and shaken (HZ-8811K bath thermostat oscillator, Deou Corporation, Changzhou, China) at 250 rpm under the predetermined temperature (25, 35, or 45 ± 1 °C) for 180 min. Following centrifugation to isolate the ARM, the clear supernatant was passed through a 0.22 μm membrane, preparing it for analysis via high-performance liquid chromatography (HPLC, Agilent 1200, Santa Clara, CA, USA). The blank control test (without the addition of ARM) and three groups of parallel experiments were set up, and the results were documented as an average. The adsorption capacity was calculated by Equation (14):

(14) $$q_{t}=\frac{(c_{0}-c_{t})\times v}{m}$$

where

*q*_t_—the adsorption capacity (mg/g);

*c*_0_—CIP concentration at initial (mg/L);

*c*_t_—CIP concentration at time *t* (mg/L);

*v*—solution volume (mL);

*m*—the mass of ARM (g).

#### 3.2.3. Response Surface Optimization

After a series of single factor experiments, adsorption temperature, solution pH, CIP initial concentration, and ARM dosage were chosen as independent variables, which were recorded as A, B, C, and D, respectively. The choice of experimental conditions for each variable was informed by initial experimental outcomes. Table S1 displays the range of independent variables and their respective levels. The adsorption efficiency was designated as the response variable *Y*. The optimization test scheme was designed by Box–Behnken RSM in Design Expert 10.7 software. The design matrix is given in Table S2.

#### 3.2.4. Adsorption Kinetics

Totals of 0.68 g of ARM and 200 mL of CIP solution at 30 mg/L concentration were combined in a 250 mL brown conical flask. The solution pH was adjusted to 3.04. Then, the flask was shaken at 250 rpm under 25~55 °C. After an interval, a certain amount of mixed liquor was withdrawn for CIP analysis.

#### 3.2.5. Adsorption Isotherm

This procedure mirrored the one outlined in Section 2.2.2, with equilibrium adsorption isotherms derived by altering CIP concentrations between 10 and 500 mg/L (pH = 3.0 ± 0.2) for a fixed amount of ARM (3.4 g/L) at temperatures (45 °C).

#### 3.2.6. Analytical Methods

CIP levels were determined using HPLC (Agilent 1200 Series, Agilent, USA), employing a reversed-phase XDB-C_18_ column (4.6 mm × 150 mm, 5 μm). The column was maintained at 30 °C, with a 10 μL sample injection volume. The eluent consisted of acetonitrile and 0.2% formic acid in a 20:80 volume ratio. The flow rate was maintained at 0.2 mL/min, and UV detection was performed at 277 nm, under which the CIP retention time (*t*_R_) was 9.768 min.

The texture, surface area, and pore size composition of samples were analyzed by SEM (JSM-6700F, JEOL, Tokyo, Japan), TEM (JEM-3010, Japan Electronics Co., Ltd., Tokyo, Japan), Barrett–Joyner–Halenda (BJH) and BET (V-Sorb 2800TP, Beijing Guoyi Precision Testing Technology Co., Ltd., Beijing, China), XRD (XRD-7000, Shimadzu, Kyoto, Japan) and XRF (PANalytical Axios, Almelo, The Netherlands). The adsorption performance of samples was analyzed by TG (TGA/DSC^3+^, Mettler Toledo, Zurich, Switzerland) and NH_3_-TPD (Bruker EMXplus, Mannheim, Germany).

The interaction of ARM and CIP at the molecular scale was investigated using ATR-FTIR analysis. The samples were recorded on a VERTEX70 instrument (Bruker, Germany).

## 4. Conclusions

RM was acidified, characterized, and used as an absorbent for removing CIP. Based on the response surface optimization model, the adsorption process was simulated, and the optimal conditions were obtained. The adsorption of CIP onto ARM adhered to a pseudo-second-order reaction (*R*^2^ = 0.999). Additionally, the adsorption isotherm fitting indicated conformity to the Langmuir–Freundlich model (*R*^2^ = 0.963). An adsorption thermodynamics analysis indicated that ARM’s adsorption of CIP was a spontaneous endothermic reaction. An ATR-FTIR analysis suggested that complex reactions existed between Al–O in ARM and the ester group –COO in CIP, and the C=O bond in CIP was likely to undergo a slight electrostatic interaction or be bound to the internal spherical surface of ARM. The results proved that ARM has potential applications in CIP removal in water.

## Figures and Tables

**Table 1:** The chemical compositions of both RM and ARM.

Samples (%)	CaO	Al_2_O_3_	SiO_2_	Na_2_O	Fe_2_O_3_	TiO_2_	K_2_O	MgO	Others
RM	26.01	23.72	17.09	11.76	11.56	5.72	1.81	0.95	1.38
ARM	40.13	8.36	10.89	2.10	28.37	4.26	2.91	1.13	1.85

**Table 2:** Constants of adsorption kinetics models.

Pseudo-First-Order Dynamic Model	Pseudo-Second-Order Dynamic Model	Intraparticle Diffusion Model		
		The First Phase	The Second Phase	The Third Phase
*k*_1_ = 0.03869 (min^−1^)	*k*_2_ = 0.023 [g/(mg·min)]	*k*_1_ = 0.40054 (min^−1^)	*k*_2_ = 0.06936 (min^−1^)	*k*_3_ = 0.02236 (min^−1^)
*q*_e_ = 4.17 (mg/g)	*q*_e_ = 7.90 (mg/g)	*C* = 6.74 (mg/g)	*C* = 1.59 (mg/g)	*C* = 1.03 (mg/g)
*R*^2^ = 0.883	*R*^2^ = 0.999	*R*^2^ = 0.861	*R*^2^ = 0.976	*R*^2^ = 0.984

**Table 3:** Parameters of adsorption isotherms for CIP adsorption onto ARM (ARM dosage: 3.4 g/L; CIP initial concentration: 10~500 mg/L; initial solution pH: 3.0; adsorption temperature: 45 °C).

Adsorption Isotherm	Fitting Curve	Parameter 1	Parameter 2	*R* ^2^
Langmuir	*Y* = 7.35866*X* + 1.39057	*K*_L_ = 0.14 (L/mg)		0.676
Freundlich	*Y* = 1.00799*X* − 1.25902	*K*_F_ = 0.28 (L/mg)	*n*_F_ = 0.99	0.999
Langmuir–Freundlich	*Y* = −1.64094*X* + 6.90526	*K*_LF_ = 0.99 (L/mg)	*n*_LF_ = 1.65	0.963

**Table 4:** Comparative assessment of CIP adsorption capacities across various adsorbents.

Adsorbents	Adsorption Capacity (mg/g)	pH	Temp (°C)	Ref.
Magnetic N-doped porous carbon	1564	7.0	25	[37]
Fe-based MOF	868.6	6.8	15	[38]
Fe-pillared clay	122.1	10	20	[39]
Chitosan/Kaolin/Fe_3_O_4_	47.85	6.0	25	[40]
Activated red mud	41.5	7.0	-	[24]
Montmorillonite	23	-	-	[41]
Schorl	8.49	5.5	-	[42]
Acidified red mud	7.84	3.0	45	This study
Kaolinite	6.31	3.5	25	[43]
Sodium alginate hydrogel	2.90	2.0	25	[44]
Sodium alginate aerogel	2.87			
AC	1.86	-	25	[45]
ZnO nanoparticles	0.16	6.0	25	[46]

**Table 5:** Thermodynamic parameter values for the adsorption.

Temperature (K)	Δ*H*^θ^ (kJ/mol)	Δ*S*^θ^ (J/mol·k)	Δ*G*^θ^ (kJ/mol)
298	0.86	281.6	−83.05
308			−85.87
318			−88.68
328			−91.50

## Data Availability

Data are contained within the article and Supplementary Materials.

## Supplementary Materials

The following supporting information can be downloaded at: https://www.mdpi.com/article/10.3390/molecules29122928/s1, Table S1: Variables and their respective ranges for the Box–Behnken experimental design; Table S2: Box–Behnken experimental design matrix; Table S3: Comparison study of TG analysis of ARM and RM sample; Table S4: Box-Behnken design real values along with observed responses for CIP adsorption capacity; Table S5: ANOVA for the quadratic equation of the response surface; Figure S1: The N_2_ adsorption/desorption isotherms for RM (a) and ARM (b); Figure S2: TGA-DSC of RM (a) and ARM (b); Figure S3: 3D surface graph and contour plot of solution pH and CIP initial concentration, 35 °C and 3.0 g/L ARM; Figure S4: Fitting curves of Pseudo-first-order model (a) and Pseudo-second-order model (b) (pH_0_ = 3.04, T = 45 °C, [CIP] = 30 mg/L) [ARM] = 3.4 g/L, r = 250 rpm; Figure S5: The efficiency of CIP leaching from different leaching solutions (pH_0_ = 3.04, T = 45 °C, [CIP] = 10~500 mg/L, [ARM] = 3.4 g/L, r = 250 rpm).

## References

[1] Jollet V., Gissane C., Schlaf M. (2014). Optimization of the neutralization of red mud by pyrolysis bio-oil using a design of experiments approach. Energy Environ. Sci..

[2] Wu J., Gong Z., Lu C., Niu S., Ding K., Xu L., Zhang K. (2018). Preparation and performance ofmodified red mud-based catalysts for selective catalytic reductionof NOx with NH_3_. Catalysts.

[3] Ren J., Chen J., Guo W., Yang B., Qin X.P., Du P. (2019). Physical, chemical, and surface charge properties of bauxite residue derived from a combined process. J. Cent. South Univ..

[4] Xue S.G., Zhu F., Kong X.F., Wu C., Huang L., Huang N., Hartley W. (2016). A review of the characterization and revegetation of bauxite residues (Red mud). Environ. Sci. Pollut. Res..

[5] Schmalenberger A., O’sullivan O., Gahan J., Cotter P.D., Courtney R. (2013). Bacterial communities established in bauxite residues with different restoration histories. Environ. Sci. Technol..

[6] Ren J., Chen J., Han L., Wang M., Yang B., Du P., Li F.S. (2018). Spatial distribution of heavy metals, salinity and alkalinity in soils around bauxite residue disposal area. Sci. Total Environ..

[7] Hua Y.M., Heal K.V., Friesl-Hanl W. (2017). The use of red mud as an immobiliser for metal/metalloid- contaminated soil: A review. J. Hazard Mater.

[8] Gu H.N., Wang N., Liu S.R. (2012). Radiological restrictions of using red mud as building material additive. Waste Manag. Res..

[9] Zhu X.B., Li W., Tang S., Zeng M.J., Bai P.Y., Chen L.J. (2017). Selective recovery of vanadium and scandium by ion exchange with D201 and solvent extraction using P507 from hydrochloric acid leaching solution of red mud. Chemosphere.

[10] Yan X.M., Miao P., Chang G.Z., Guo Q.J. (2018). Characteristics of microstructures and reactivities during steam gasification of coal char catalyzed by red mud. Chem. Ind. Eng. Prog..

[11] Wang Y., Yu Y.G., Li H.Y., Shen C.C. (2016). Comparison study of phosphorus adsorption on different waste solids: Fly ash, red mud and ferric-alum water treatment residues. J. Environ. Sci..

[12] Li C.M., Yu J., Li W.S., He Y., Qiu Y.L., Li P., Wang C., Huang F.L., Wang D.L., Gao S.Q. (2018). Immobilization, enrichment and recycling of Cr(Ⅵ) from wastewater using a red mud/carbon material to produce the valuable chromite (FeCr_2_O_4_). Chem. Eng. J..

[13] Wang Y.Z., Zhang L.Y., Yan Z.W., Shao L.H., Kang H., Wei G.T. (2015). Application of a low-cost bagasse carbon-red mud (BCRM) adsorbent for adsorption of methylene blue cationic dye: Adsorption performance, kinetics, isotherm, and thermodynamics. Desalination Water Treat..

[14] Ye J., Cong X.N., Zhang P.Y., Hoffmann E., Zeng G.M., Liu Y., Fang W., Wu Y., Zhang H.B. (2015). Interaction between phosphate and acid-activated neutralized red mud during adsorption process. Appl. Surf. Sci..

[15] Walsh C. (2003). Antibiotics: Actions, Origins, Resistance.

[16] Kolpin D.W., Furlong E.T., Meyer M. (2002). Pharmaceuticals, hormones, and other organic wastewater contami- nants in U.S.streams, 1999–2000: A national reconnaissance. Environ. Sci. Technol..

[17] Martins A.F., Vasconcelos T.G., Henriques D.M., Frank C.S. (2008). Concentration of ciprofloxacin in Brazilian hospital effluent and preliminary risk assessment: A case study. Clean-Soil Air Water.

[18] Orimolade B.O., Oladipo A.O., Idris A.O., Usisipho F., Azizi S., Maaza M., Lebelo S.L., Mamba B.B. (2023). Advancements in electrochemical technologies for the removal offluoroquinolone antibiotics in wastewater: A review. Sci. Total Environ..

[19] Ni F., He J.S., Wang Y.B., Luan Z.K. (2015). Preparation and characterization of a cost-effective red mud/polyaluminum chloride composite coagulant for enhanced phosphate removal from aqueous solutions. J. Water Process Eng..

[20] Atasoy A. (2005). An investigation on characterization and thermal analysis of the aughinish red mud. J. Therm. Anal. Calorim..

[21] Huang W.W., Wang S.B., Zhu Z.H., Li L., Yao X.D., Rudolph V., Haghseresht F. (2008). Phosphate removal from wastewater using red mud. J. Hazard. Mater..

[22] Jayasankar K., Ray P.K., Chaubey A.K., Padhi A., Satapathy B.K., Mukherjee P.S. (2012). Production of pig iron from red mud waste fines using thermal plasma technology. Int. J. Miner. Metall. Mater..

[23] Manoj K.S., Sandip M., Saswati S.D., Pranati B., Raj K.P. (2013). Removal of Pb(II) from aqueous solution by acid activated red mud. J. Environ. Chem. Eng..

[24] Balarak D., Joghataei A., Mostafapour F.K., Bazrafshan E., Pharm J. (2017). Ciprofloxacin antibiotics removal from effluent using heat-acid activated Red Mud. J. Pharm. Res. Int..

[25] Zhilkina A.V., Gordienko A.A., Prokudina N.A., Trusov L.I., Kuz’micheva G.M., Dulina N.A., Savinkina E.V. (2013). Determination of the size of particles of highly dispersed materials by low temperature nitrogen adsorption. Russ. J. Phys. Chem. A.

[26] Guru S., Amritphale S.S., Mishra J., Joshi S. (2019). Multicomponent red mud-polyester composites for neutron shielding Application. Mater. Chem. Phys..

[27] Fang H., Liang W., Ma C., Tao Q., Liu J. (2023). Effect of interaction between Pd and Fe in modified red mud on catalytic decomposition of toluene. Environ. Sci. Pollut. Res..

[28] Deihimi N., Irannajad M., Rezai B. (2018). Characterization studies of red mud modification processes as adsorbent for enhancing ferricyanide removal. J. Environ. Manag..

[29] Liang W.J., Tao Q.Y., Fang H.P., Zhang C.H., Liu J., Bin F., Kang R.N. (2024). Modification of red mud catalyst using oxalic acid-assisted UV treatment for toluene removal. Catal. Today.

[30] Zhang J., Hayat K., Zhang X.M., Tong J.M., Xia S.Q. (2010). Separation and purification of flavonoid from ginkgo extract by polyamide resin. Sep. Sci. Technol..

[31] Liu Y., Lin C., Wu Y. (2007). Characterization of red mud derived from a combined Bayer process and bauxite calcination method. J. Hazard. Mater..

[32] Zhu X., Li W., Guan X. (2015). An active dealkalization of red mud with roasting and water leaching. J. Hazard. Mater..

[33] Liang W., Zhu Y., Ren S., Li Q., Song L., Shi X. (2021). Catalytic combustion of chlorobenzene at low temperature over Ru-Ce/TiO_2_: High activity and high selectivity. Appl. Catal. A Gen..

[34] Saha S., Sarkar P. (2012). Arsenic remediation from drinking water by synthesized nano-alumina dispersed in chitosan-grafted polyacry- lamide. J. Hazard. Mater..

[35] Hu X.J., Wang J.S., Liu Y.G., Li X., Zeng G.M., Bao Z.L., Zeng X.X., Chen A.W., Long F. (2011). Adsorption of chromium (VI) by ethylenediamine-modified cross-linked magnetic chitosan resin: Isotherms, kinetics and thermodynamics. J. Hazard. Mater..

[36] Ofomaja A.E. (2008). Kinetic study and sorption mechanism of methylene blue and methyl violet onto mansonia (*Mansonia altissima*) wood sawdust. Chem. Eng. J..

[37] Tang Y., Chen Q., Li W., Xie X., Zhang W., Zhang X., Chai H., Huang Y. (2020). Engineering magnetic N-doped porous carbon with super-high ciprofloxacin adsorption capacity and wide pH adaptability. J. Hazard. Mater..

[38] Jiang C., Zhang X., Xu X., Wang L. (2016). Magnetic mesoporous carbon material with strong ciprofloxacin adsorption removal property fabricated through the calcination of mixed valence Fe based metal-organic framework. J. Porous Mater..

[39] Roca Jalil M.E., Baschini M., Sapag K. (2017). Removal of Ciprofloxacin from aqueous solutions using pillared clays. Materials.

[40] Ma W., Dai J., Dai X., Yan Y. (2014). Preparation and characterization of Chitosan/Kaolin/Fe_3_O_4_ magnetic microspheres and their application for the removal of Ciprofloxacin. Adsorpt. Sci. Technol..

[41] Chen H., Gao B., Yang L.Y., Ma L.Q. (2015). Montmorillonite enhanced ciprofloxacin transport in saturated porous media with sorbed ciprofloxacin showing antibiotic activity. J. Contam. Hydrol..

[42] Yin D., Xu Z., Shi J., Shen L., He Z. (2018). Adsorption characteristics of ciprofloxacin on the schorl: Kinetics, thermodynamics, effect of metal ion and mechanisms. J. Water Reuse Desalination.

[43] Li Z.H., Hong H.L., Liao L.B., Ackley C.J., Schulz L.A., MacDonald R.A., Mihelich A.L., Emard S.M. (2011). A mechanistic study of ciprofloxacin removal by kaolinite. Colloids Surf. B Biointerfaces.

[44] Yu F., Li Y., Han S., Ma J. (2016). Adsorptive removal of ciprofloxacin by sodium alginate/graphene oxide composite beads from aqueous solution. J. Colloid Interface Sci..

[45] Avcı A., Inci I., Baylan N. (2019). A comparative adsorption study with various adsorbents for the removal of Ciprofloxacin hydrochloride from water. Water Air Soil Pollut..

[46] Dhiman N., Sharma N. (2019). Batch adsorption studies on the removal of ciprofloxacin hydrochloride from aqueous solution using ZnO nanoparticles and groundnut (*Arachis hypogaea*) shell powder: A comparison. Indian Chem. Eng..

[47] Chen T., Da T., Ma Y. (2021). Reasonable calculation of the thermodynamic parameters from adsorption equilibrium constant. J. Mol. Liq..

[48] Reza R.A., Ahmaruzzaman M., Sil A.K., Gupta V.K. (2014). Comparative adsorption behavior of ibuprofen and clofibric acid onto microwave assisted activated bamboo waste. Ind. Eng. Chem. Res..

[49] Kumar R., Rashid J., Barakat M.A. (2014). Synthesis and characterization of a starch-AlOOH-FeS_2_ nanocomposite for the adsorption of congo red dye from aqueous solution. RSC Adv..

[50] Venkatesan G., Narayanan S.L. (2018). Synthesis of Fe_2_O_3_-coated and HCl-treated bauxite ore waste for the adsorption of arsenic (Ⅲ) from aqueous solution: Isotherm and kinetic models. Chem. Eng. Commun..

[51] Castaldia P., Silvetti M., Enzob S., Melis P. (2010). Study of sorption processes and FT-IR analysis of arsenate sorbed onto red muds (a bauxite ore processing waste). J. Hazard. Mater..

[52] Paras T., Dharni V. (2007). Spectroscopic investigation of ciprofloxacin speciation at the goethite-water interface. Environ. Sci. Technol..

