# Supplementary material for: Ciprofloxacin Removal via Acid-Modified Red Mud: Optimizing the Process, Analyzing the Adsorption Features, and Exploring the Underlying Mechanism
Source: Molecules. 2024 Jun 20;29(12):2928. doi: 10.3390/molecules29122928 (PMC11207061; doi:10.3390/molecules29122928)
Supplement: Supplementary file 1 [file molecules-29-02928-s001.zip › molecules-3041807-supplementary.pdf]

Supporting Information for

Ciprofloxacin Removal via Acid-Modified Red Mud: Optimizing the Process, Analyzing the Adsorption Features, and Exploring the Underlying Mechanism

Jingzhuan Shi[1]*,Wanqiong Wang[1],Ziyi Li[1],Yingjuan Shi[2]

[1]School of Chemistry and Environmental Science, Shaanxi University of Technology, Han zhong, 723001, China

[2]Shaanxi Reconnaissance Design & Research Institute of Water Environmental Engineering, Xi'an 710021, China

*Corresponding author: shijingzhuan@snut.edu.cn

This supporting information contains 10 figures and tables.

Table S1. Variables and their respective ranges for the Box–Behnken experimental design.

Control factors	Unit	Symbol	Real values and coded levels		
			Low (-1)	Mid (0)	High (1)
Adsorption temperature	℃	A	25	35	45
Solution pH		B	3	7	11
CIP initial concentration	mg/L	C	10	20	30
ARM dosage	g/L	D	3	4	5

Table S2. Box–Behnken experimental design matrix.

Number	A(℃)	B	C(mg/L)	D(g/L)	Number	A(℃)	B	C(mg/L)	D(g/L)
1	25	11	20	4	16	25	7	20	3
2	35	7	30	3	17	25	7	10	4
3	25	3	20	4	18	35	3	20	5
4	35	7	10	5	19	35	7	30	5
5	35	7	20	4	20	45	11	20	4
6	45	7	20	5	21	35	7	10	3
7	35	3	10	4	22	25	7	30	4
8	25	7	20	5	23	35	7	20	4
9	35	11	30	4	24	35	7	20	4
10	35	3	30	4	25	35	11	20	5
11	45	7	20	3	26	35	7	20	4
12	45	7	30	4	27	35	7	20	4
13	45	7	10	4	28	35	11	10	4
14	35	11	20	3	29	45	3	20	4
15	35	3	20	3					

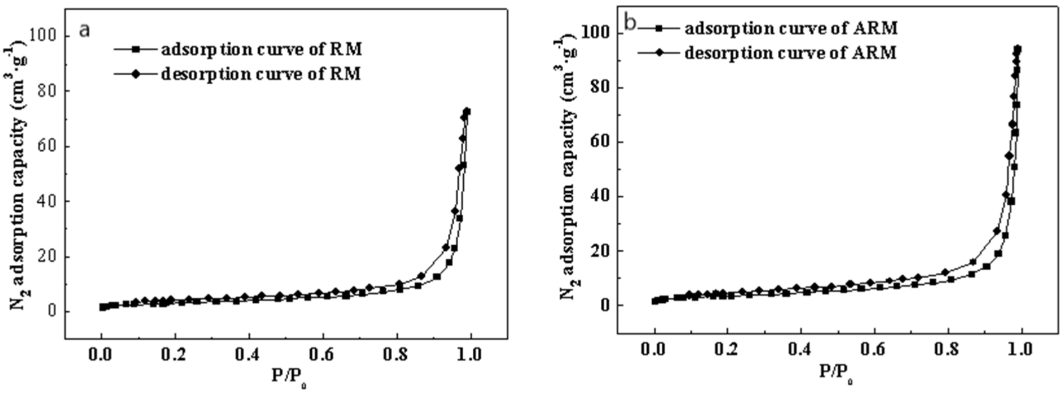

Figure S1. The N_2 adsorption/desorption isotherms for RM(a) and ARM(b).

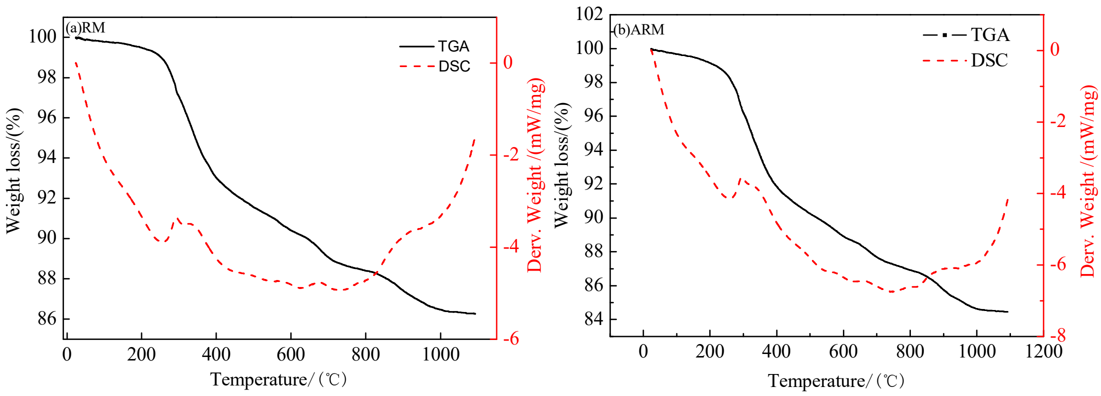

Figure S2 . TGA-DSC of RM(a) and ARM(b)

Table S3. Comparison study of TG analysis of ARM and RM sample

Sample	Mass loss(%)	Temperature(℃)	References
Red mud (Zhengzhou Changcheng)	3	20~150	[1]
	3.6	105~450	
	4.3	560~720	
Total	10.9	20~880	
Red mud (Shanxi Yuncheng)	3	25~300	In this present study
	6.6	300~600	
	4.1	600~1100	
Total	13.73	24~1100	
Acidified red mud (Shanxi Yuncheng)	3.8	25~300	In this present study
	7.3	300~600	
	4.4	600~1100	
Total	15.55	24~1100	

Table S4. Box-Behnken design real values along with observed responses for CIP adsorption capacity.

Number	Adsorption capacity（mg/g）		Number	Adsorption capacity（mg/g）	
	Actual value	Predicted value		Actual value	Predicted value
1	2.35	2.34	16	2.99	3.08
2	4.07	4.47	17	1.28	1.02
3	4.78	4.90	18	3.82	4.08
4	1.04	0.72	19	2.76	2.89
5	2.34	2.34	20	2.18	2.24
6	1.94	1.74	21	1.66	1.62
7	2.28	2.77	22	3.21	3.53
8	2.34	2.09	23	2.34	2.34

Number	Adsorption capacity（mg/g）		Number	Adsorption capacity（mg/g）	
	Actual value	Predicted value		Actual value	Predicted value
9	3.32	2.72	24	2.34	2.34
10	7.23	6.70	25	1.85	2.22
11	3.11	3.24	26	2.34	2.34
12	3.16	3.44	27	2.34	2.34
13	1.22	0.93	28	1.21	1.63
14	2.99	2.76	29	4.78	4.80
15	6.37	6.02			

Table S5. ANOVA for the quadratic equation of the response surface.

Source	Sum of squares	d_f	Mean square	F-value	P-value
Model	56.73	11	5.16	40.08	< 0.0001
A	0.026	1	0.026	0.20	0.6595
B	19.66	1	19.66	152.77	< 0.0001
C	18.90	1	18.90	146.88	< 0.0001
D	4.61	1	4.61	35.83	< 0.0001
AD	0.067	1	0.067	0.52	0.4814
BC	2.01	1	2.01	15.66	0.0010
BD	0.50	1	0.50	3.89	0.0651
CD	0.11	1	0.11	0.88	0.3606
B2	10.18	1	10.18	79.12	< 0.0001
C2	0.084	1	0.084	0.65	0.4315
D2	0.27	1	0.27	2.09	0.1662
Residual	2.19	17	0.13		
Lack of Fit	2.19	13	0.17		
Pure Error	0.000	4	0.000		
Cor Total	58.92	28			

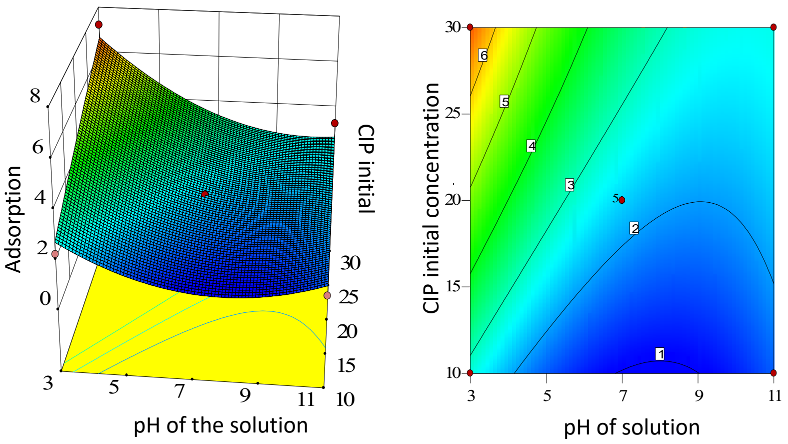

Figure S3. 3D surface graph and contour plot of solution pH and CIP initial concentration, 35°C and 3.0 g/L ARM.

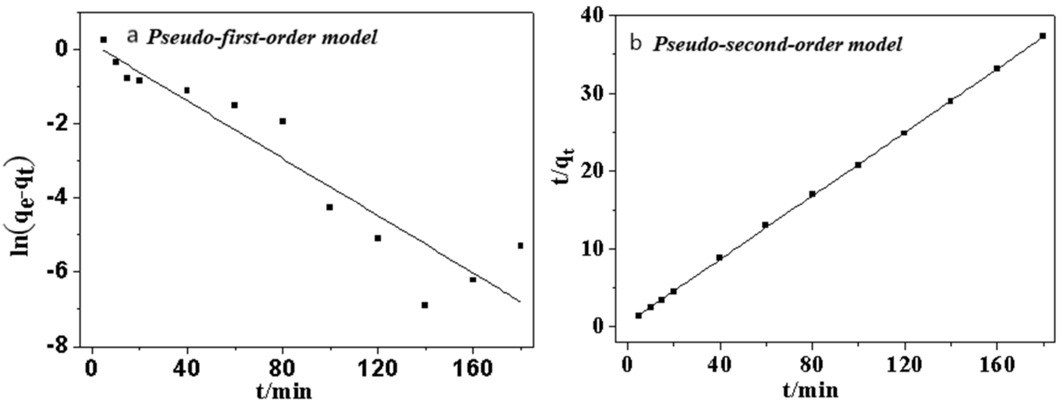

Figure S4. Fitting curves of Pseudo-first-order model(a) and Pseudo-second-order model(b)(pH_0=3.04, T=45℃, [CIP]=30 mg/L)[ARM]=3.4 g/L, r=250 rpm)

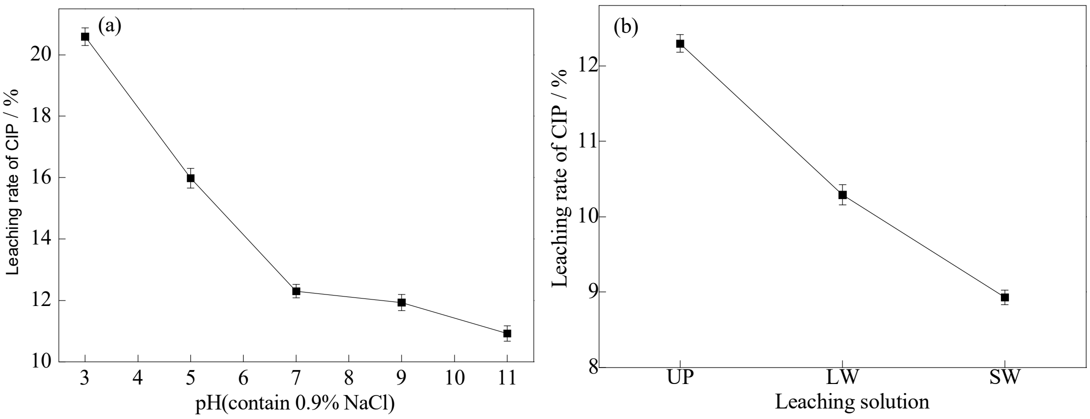

Figure S5. The efficiency of CIP leaching from different leaching solutions (pH_0=3.04, T=45℃, [CIP]=10~500 mg/L, [ARM]=3.4 g/L, r=250 rpm).

References

[1] Liu, Y.; Lin, C.; Wu, Y. Characterization of red mud derived from a combined Bayer process and bauxite calcination method. *J. Hazard. Mater.* **2007**, *146*, 255–261.
